# Dengue Virus Infections among Peace Corps Volunteers in Timor-Leste, 2018–2019

**DOI:** 10.4269/ajtmh.21-0020

**Published:** 2021-04-26

**Authors:** Liliana Sánchez-González, Margaret Venuto, Scott Poe, Chelsea G. Major, Leonardus Baskara, Sevinj Abdiyeva, Daniel Murphy, Jorge L. Munoz-Jordan, Freddy A. Medina, Gabriela Paz-Bailey, Kyle Petersen, Karen Becker, Tyler M. Sharp

**Affiliations:** 1 Dengue Branch, Centers for Disease Control and Prevention, San Juan, Puerto Rico;; 2 Epidemiology and Surveillance Unit, Office of Health Services, United States Peace Corps, Washington, District of Columbia;; 3 Timor-Leste Country Office, Office of Health Services, U.S. Peace Corps, Washington, District of Columbia;; 4 U.S. Public Health Service, Rockville, Maryland

## Abstract

Dengue is an ongoing health risk for Peace Corps Volunteers (PCVs) working in the tropics. On May 2019, the Peace Corps Office of Health Services notified the Centers for Disease Control and Prevention (CDC) of a dengue outbreak among PCVs in Timor-Leste. The purpose of this investigation was to identify the clinical, demographic, and epidemiological characteristics of PCVs with dengue and recommend dengue preventive measures. To identify PCVs with dengue and describe disease severity, the medical records of PCVs reporting fever during September 2018–June 2019 were reviewed. To identify factors associated with dengue virus (DENV) infection, we administered a questionnaire on demographics, travel history, and mosquito avoidance behaviors and collected blood specimens to detect the anti-DENV IgM antibody to diagnose recent infection. Of 35 PCVs in-country, 11 (31%) tested positive for dengue (NS1, IgM, PCR), eight requiring hospitalization and medical evacuation. Among 27 (77%) PCVs who participated in the investigation, all reported having been recently bitten by mosquitoes and 56% reported being bitten most often at home; only 16 (59%) reported having screens on bedroom windows. Nearly all (93%) PCVs reported using a bed net every night; fewer (70%) reported using mosquito repellent at least once a day. No behaviors were significantly associated with DENV infection. Raising awareness of dengue risk among PCVs and continuing to encourage mosquito avoidance behavior to prevent dengue is critical. Access to and use of measures to avoid mosquito bites should be improved or implemented. Peace Corps medical officers should continue to receive an annual refresher training on dengue clinical management.

## BACKGROUND

Dengue is the most common arboviral disease globally and remains a risk for both residents of and travelers to tropical and subtropical regions.^1^ Most dengue virus (DENV) infections are asymptomatic,^2^ and common signs and symptoms among symptomatic patients include fever, myalgia, arthralgia, rash, and minor hemorrhagic manifestations (e.g., petechiae, gingival bleeding).^3^ Diagnosis of dengue is complicated by the overlap of its clinical manifestations with those of other acute febrile illnesses including other arboviral diseases (e.g., chikungunya, Zika) and by limited access to laboratory testing. Roughly 5% of patients may progress to severe dengue, which is characterized by increased vascular permeability leading to shock, hemorrhage, and, in some cases, death.^3^ Severe dengue is often preceded by warning signs (e.g., abdominal pain, lethargy, and hepatomegaly).^4,5^ Early recognition of warning signs and appropriate clinical management are key to improving clinical outcomes among dengue patients and have been shown to reduce the case-fatality rate among dengue patients from ≈5% to < 0.5%.^6^

Dengue is endemic in Timor-Leste where mosquito vectors *Aedes aegypti* and *Aedes albopictus* are present, and multiple epidemics have occurred over the past two decades.^7,8^ Because of poor access to and use of health care, high case-fatality rates (i.e., 12%) have been reported during prior epidemics.^9,10^ During January–April 2019, the World Health Organization (WHO) office in Timor-Leste reported an increase in the number of cases of dengue (*n* = 532) compared with the same period in the previous year (*n* = 317)^11^ and 5 deaths, consistent with a dengue epidemic.^7^

The Peace Corps, an independent U.S. government agency, assigns volunteers (PCVs) to 64 developing countries to work on different projects including education, health, agriculture, and environment. PCVs typically serve 27 months at posts in rural areas with limited infrastructure and increased risk of exposure to various communicable diseases. Because they usually live with local families in areas that are often isolated and lack vector control programs, the risk of dengue among PCVs is high. Among dengue endemic countries where PCVs were serving between 2000 and 2014, incidence of dengue was highest for those in Timor-Leste (11 cases per 1,000 volunteer-months).^12^

On May 6, 2019, CDC was notified by the Peace Corps Office of Health Services of a recent increase in dengue cases among PCVs in Timor-Leste. Because of the ongoing dengue epidemic exacerbated by limited access to adequate medical care in-country, the Peace Corps requested assistance from CDC to identify and gain a better understanding of factors associated with DENV exposure among PCVs and to provide recommendations to mitigate the risk of exposure among PCVs serving in Timor-Leste and the community.

The objectives of this investigation were to: 1) describe the clinical characteristics and outcomes among PCVs with febrile illness during the dengue outbreak in Timor-Leste in 2018–2019; 2) identify clinical or demographic characteristics associated with PCVs with dengue; 3) identify risk factors associated with infection with DENV or other arboviruses; and 4) recommend prevention and control measures to protect PCVs serving in Timor-Leste from arboviral infection.

## METHODS

### Participant recruitment and data collection.

The investigation consisted of two parts: 1) Review of medical records for PCVs who had sought medical care for fever or suspicion of dengue during September 2018–June 2019 (referred to as “medical records review”) and 2) A survey and blood collection for additional testing (referred to as “survey and blood sample collection”).

First, for the medical records review, for any PCVs in Timor-Leste who sought medical care for an acute febrile illness or dengue suspicion during September 2018–June 2019, clinical data and DENV laboratory results were abstracted from medical records in the Peace Corps Medical Electronic Documentation and Inventory Control System (PCMEDICS). These data were evaluated using the WHO 2009 dengue case definitions.^1^

For the second part, the survey and blood sample collection, with the support of Peace Corps medical officers, PCVs in-country at the time of these activities (*n* = 35) were provided an overview on the purpose and procedures of this part of the investigation and invited to participate. Questionnaires were completed and data samples collected between July 13th and July 19th, 2019. PCVs who agreed to participate: 1) completed a one-time self-administered questionnaire on demographics, recent illness history, vaccinations, clinical care, and mosquito-bite prevention behaviors during the previous six months, October 2018–June 2019, and 2) provided a blood specimen for diagnostic testing to detect recent infection with DENV, chikungunya virus (CHIKV), and Zika virus (ZIKV). All PCVs were serving in Timor-Leste for the complete period of interest for this investigation. Diagnostic testing was performed at the request of the medical officers, who returned test results to PCVs.

### Definitions.

For the medical record review, we defined “dengue patients” as PCVs who sought medical care for acute febrile illness or other acute symptoms and had laboratory diagnostic evidence of DENV infection (i.e., positive result for DENV NS1, anti-DENV IgM, or DENV RT-PCR).

For the survey and blood sample collection, we defined “participants” as PCVs in Timor-Leste at the time of the investigation who answered the questionnaire and provided a blood sample. Participants with previously documented DENV infection through the medical records review and/or through diagnostic testing performed at the CDC Dengue Branch are included in the DENV infection group for the survey and blood sample collection analysis.

### Diagnostic testing.

For PCVs identified through the medical records review who didn’t participate in the survey and blood sample collection, no additional testing was performed. Serum specimens of the participants of the survey and blood sample collection were stored at −20°C until being shipped on dry ice to the CDC Dengue Branch in San Juan, Puerto Rico, where they were stored at −70°C. Specimens were tested by IgM ELISA to detect evidence of recent infection with DENV,^13^ ZIKV,^14^ or CHIKV.^15^ Specimens positive for anti-ZIKV and anti-CHIKV IgM antibody by ELISA were tested by plaque reduction neutralization assay (PRNT). Testing plan included PRNT for any positive anti-DENV specimen from participants without a previous viral test for confirmation. RT-PCR to detect DENV, CHIKV, and ZIKV nucleic acid was not performed because all participants were asymptomatic at the time of specimen collection.

### Data analysis.

For the medical record review, we used medical record data to compare demographic and clinical characteristics of dengue patients to those people who sought care for an acute febrile illness without laboratory diagnostic evidence of dengue. For the survey and blood sample collection, we used questionnaire responses to compare participants with and without laboratory diagnostic evidence of DENV infection to identify risk factors associated with DENV infection among PCVs. Pearson’s χ^2^ test and Fisher’s exact test were used to compare categorical variables between groups. Median 2-sample test was used to compare continuous variables. For all tests, *P* values less than 0.05 were considered statistically significant. Data analyses were performed using SAS 9.4 (Cary, NC) statistical software.

### Human subjects.

CDC’s Human Subjects Office reviewed this investigation´s protocol and provided a nonresearch determination because it was considered part of a public health response. Participation was voluntary, and responses to survey questions were confidential. Identifiable data from questionnaires was not shared with Peace Corps medical officers or Headquarters staff. All participants were guaranteed that their answers, including compliance with dengue prevention measures, would not have any negative impact. All participants provided written consent. PCVs provided permission to the Peace Corps to access medical records.

The presentation and discussion of findings follow the Strengthening the Reporting of Observational Studies in Epidemiology (STROBE) reporting guidelines.

## RESULTS

### Medical records review—Clinical characteristics and outcomes among PCVs with febrile illness or suspicion of dengue (*n* = 20).

PCVs typically receive their medical care from PC medical officers first, who then refer them to local hospitals when needed. Through review of medical records in PCMEDICS, 20 PCVs were identified who had sought medical care through their PC Medical Office for acute febrile illness or suspicion of dengue during September 2018–June 2019 among the 35 PCVs in-country. Dengue diagnostic testing had been performed for all during their medical care using the Dengue Duo (Standard Diagnostics, Inc.) test, which detects DENV NS1 antigen and anti-DENV IgM antibody. Five (25%) PCVs were also tested by RT-PCR to detect DENV nucleic acid.

In total, 11 (55%) of the 20 PCVs with acute febrile illness or suspicion of dengue had laboratory evidence of DENV infection. Laboratory tests for DENV diagnosis were conducted according to days post-onset (DPO) and availability in local and referral hospitals. Typically, patients managed in Timor-Leste were tested by the rapid NS1/IgM assay, whereas those people who were med-evacuated to Thailand were tested by RT-PCR. Median of DPO testing was 1.5 days (range 0–9 days). Testing for dengue NS1, IgM, and IgG was conducted among all 20 PCVs. NS1 was detected among 10 PCVs (Median DPO = 1, range 0–6), of which four were also positive for anti-DENV IgM antibody (Median DPO = 2.5, range 0–6). Five PCVs were positive for DENV RNA by RT-PCR (Median DPO = 1, range 1–3): one was negative for NS1, three were positive for NS1, and one was positive for NS1 and anti-DENV IgM antibody. Among PCVs that tested positive by RT-PCR, DENV-1 (*n* = 2), and DENV-3 (*n* = 3), serotypes were detected.

We compared demographic and clinical characteristics of dengue patients to those who sought care for an acute febrile illness and had no laboratory diagnostic evidence of dengue (Table 1). Median age among all patients was 24 years (range: 22–25) and half (50%) were female. Nausea, vomiting, and headache were common among all patients, and all dengue patients presented with fever and myalgia. Rash was significantly more common in dengue patients (73% versus 11%, respectively; *P* < 0.05). No other signs and symptoms or demographic characteristics were significantly associated with dengue patients.

**Table 1:** Characteristics of Peace Corps volunteers in Timor-Leste with fever or clinical suspicion of dengue by status of dengue virus infection, September 2018–June 2019 (*n* = 20)

	All patients with AFI or dengue suspicion, *n* = 20 (%)	Patients with AFI or dengue suspicion and evidence of DENV infection, *n* = 11 (%)	Patients with AFI or dengue suspicion and no evidence of DENV infection, *n* = 9 (%)	*P* value*
Demographic characteristics				
Age in years, median (range)	24 (22–45)	26 (22–45)	23 (22–27)	0.272
Female gender	10 (50)	7 (64)	3 (33)	0.369
Signs and symptoms, *n* (%)				
Fever	19 (95)	11 (100)	8 (89)	0.450
Nausea or vomiting	14 (70)	8 (73)	6 (67)	1.000
Persistent vomiting	3 (15)	2 (18)	1 (11)	1.000
Rash	9 (45)	8 (73)	1 (11)	**0.009**
Headache	13 (65)	8 (73)	5 (56)	0.642
Eye pain	3 (15)	3 (27)	0 (0)	0.218
Arthralgia	3 (15)	3 (27)	1 (11)	0.218
Myalgia	17 (85)	11 (100)	6 (67)	0.073
Abdominal pain/tenderness	3 (15)	1 (9)	2 (22)	0.565
Diarrhea	5 (25)	1 (9)	4 (44)	0.127
Cold skin/cold extremities	1 (5)	1 (9)	0 (0)	1.000
Mottled skin	1 (5)	1 (9)	0 (0)	1.000

* *P* < 0.05 = statistically significant.

Dengue warning signs were reported in three dengue patients. One patient who tested positive for DENV-3 by RT-PCR had persistent vomiting and a pulse pressure < 20. The other two patients, who tested positive for DENV by NS1, presented with bleeding (menorrhagia) and persistent vomiting, as well as abdominal pain and ascites, respectively. Clinical findings among dengue patients also included hypotension and marked thrombocytopenia, but none met the case definitions for severe dengue or dengue hemorrhagic fever.

**Figure 1.**
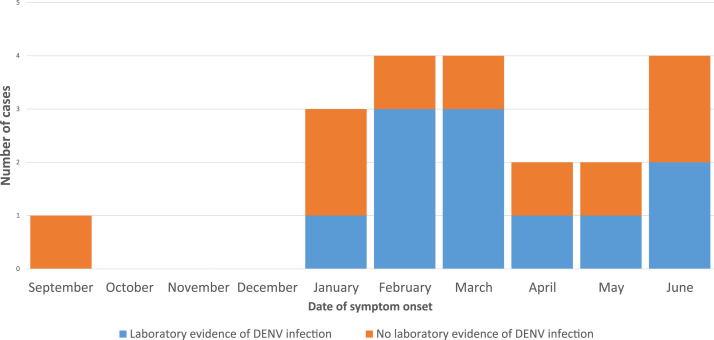
Laboratory evidence of dengue among Peace Corps volunteers in Timor-Leste who sought medical care for acute febrile illness or suspicion of dengue during September 2018 to June 2019 (*n* = 20). This figure appears in color at www.ajtmh.org.

The first patient with laboratory diagnostic evidence of DENV infection had symptom onset in January 2019. Additional cases were identified through June 2019 (Figure 1). The greatest numbers of monthly dengue patients were identified in February and March (*n* = 3).

### Survey and blood sample collection—Factors associated with DENV infection (*n* = 27).

**Figure 2.**
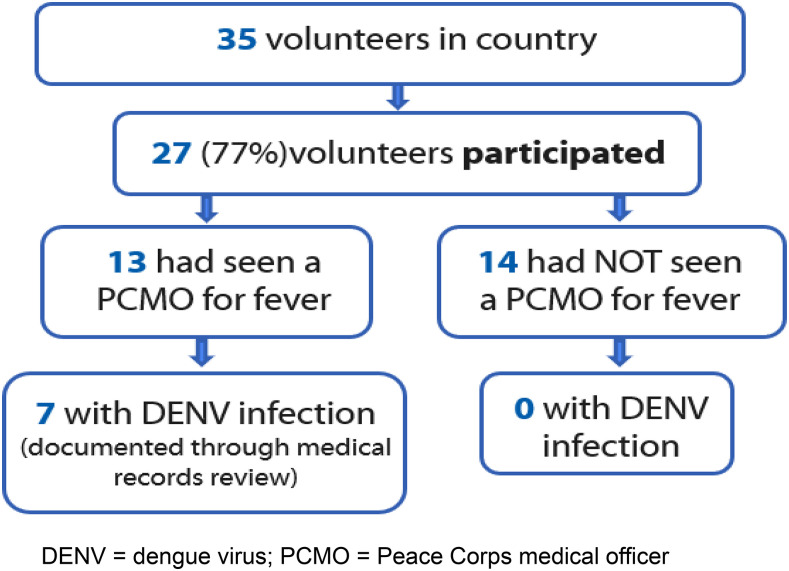
Overall participation of Peace Corps volunteers in a survey to identify factors associated with dengue virus infection, Timor-Leste, 2019. This figure appears in color at www.ajtmh.org.

**Figure 3.**
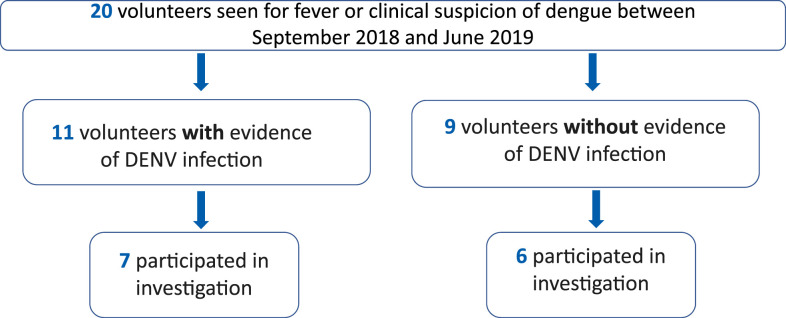
Participation of Peace Corps volunteers identified through the medical records review in a survey to identify factors associated with dengue virus infection, Timor-Leste, 2019. This figure appears in color at www.ajtmh.org.

Among 35 PCVs in Timor-Leste at the time of the investigation, 27 (77%) participated in the survey and blood sample collection aspect of this investigation. This includes 13 out of the 20 PCVs identified in the medical records review previously described (Figures 2 and 3). Among all participants, 17 (63%) were male, and median age was 25 years (range: 22–67). Most participants reported having been vaccinated against Japanese encephalitis (JE) (*n* = 21, 78%) and 7 (26%) against yellow fever (YF). Seven (26%) PCVs reported having lived in a dengue-endemic area before moving to Timor-Leste. Two (7%) reported having been diagnosed with dengue prior to arriving in Timor-Leste.

Anti-DENV IgM antibody was detected in serum specimens from two (7%) participants, both of whom had been diagnosed with dengue in 2019 and identified in the first section of this paper through the medical records review. As these participants had a positive viral test documented in their medical record, PRNT testing for DENV was not conducted. Anti-ZIKV IgM antibody was detected in one PCV for whom anti-DENV IgM antibody was not detected; confirmatory testing by PRNT did not detect neutralizing anti-ZIKV or -DENV antibodies. As the PCV reported having been recently vaccinated against JE, the positive anti-ZIKV IgM result may have been a result of cross reactivity. Anti-CHIKV IgM antibody was detected in another PCV; confirmatory testing by PRNT did not detect neutralizing anti-CHIKV antibodies.

For this analysis, participants with previously documented DENV infection through the medical records review and/or through diagnostic testing performed at the CDC Dengue Branch were included in the DENV infection group (*n* = 7) (Figure 3). No significant differences by DENV infection status were identified by demographic characteristics, vaccination history, municipality of residence, living situation, work type, work conditions, or nights spent outside the municipality of residence (Supplemental Table 1).

A total of 22 (81%) participants reported at least one episode of fever during October 2018–June 2019, including all participants with evidence of DENV infection (Table 2). The only symptom significantly associated with DENV infection was myalgia (*P* < 0.05). Duration of illness was significantly longer among PCVs with evidence of DENV infection compared with those people without evidence of DENV infection (median: 10 versus 5 days; *P* < 0.05). Among 22 PCVs who reported fever, 15 (68%) were evaluated by their Peace Corps medical officer. Of these, seven (47%) were diagnosed with dengue, all of whom had evidence of DENV infection. Of these, 5 (71%) were hospitalized and required medical evacuation. The need for medical evacuation was determined by the attending physician; and given resources limitations in-country, patients who need hospitalization or presenting with worsening symptoms were evacuated.

**Table 2:** Clinical characteristics of Peace Corps volunteers in Timor-Leste reporting illness episodes in October 2018–June 2019 by status of dengue virus infection (*n* = 27)

	All participants, *n* = 27 (%)	Participants with evidence of DENV infection, *n* = 7 (%)	Participants without evidence of DENV infection, *n* = 20 (%)	*P* value*
Demographic characteristics				
Age in years, median (range)	25 (22–67)	26 (23–45)	25 (22–67)	0.4210
Female gender, *n* (%)	10 (37)	4 (57)	6 (30)	0.3648
Acute febrile illness	**22 (81)**	**7 (100)**	**15 (75)**	0.2834
Nausea or vomiting†	9 (41)	3 (43)	6 (40)	1.0000
Rash†	4 (18)	3 (43)	1 (7)	0.0766
Headache†	21 (95)	7 (100)	14 (93)	1.0000
Eye pain†	4 (18)	2 (29)	2 (13)	0.5646
Arthralgia†	3 (14)	2 (29)	1 (7)	0.2273
Myalgia†	9 (41)	6 (86)	3 (20)	**0.0066**
Abdominal pain or tenderness†	5 (23)	1 (14)	4 (27)	1.0000
Diarrhea†	10 (45)	2 (29)	8 (53)	0.3808
Runny nose†	7 (32)	2 (29)	5 (33)	1.0000
Sore throat†	8 (36)	2 (29)	6 (40)	1.0000
Red eyes†	1 (5)	1 (14)	0 (0)	0.3182
Calf pain†	1 (5)	1 (14)	0 (0)	0.3182
Red or swollen joints†	1 (5)	1 (14)	0 (0)	0.3182
Illness duration in days, median† (range)	5 (2–30)	10 (8–17)	5 (2–30)	**0.0007**

* *P* < 0.05 = statistically significant.

† Denominator excludes individuals who did not report an acute febrile illness (*n* = 5). All calculations are based on participants who reported an acute febrile illness (*n* = 22) with evidence of DENV infection (*n* = 7) and without evidence of DENV infection (*n* = 15).

All participants reported being bitten by mosquitoes during the investigation period, and most (*n* = 17, 63%) reported being bitten most often during the evening (Table 3). Most participants reported being bitten most often at home (*n* = 14, 56%) and sleeping under a bed net every night (*n* = 25, 93%). No PCVs reported having used permethrin to treat clothes before or after arrival in Timor-Leste. Most PCVs (*n* = 16, 59%) also reported having screens on the windows of their bedroom (Table 3), and roughly one-third (*n* = 8, 30%) reported daily use of mosquito repellent. Most PCVs (*n* = 24, 89%) recalled completing training on mosquito avoidance, and 17 (63%) and 15 (56%) reported knowing that DENV was transmitted by mosquitoes and the risk of exposure to dengue in Timor-Leste, respectively. No reported mosquito avoidance behaviors or knowledge were significantly associated with DENV infection.

**Table 3:** Mosquito bite prevention measures and mosquito bites-related characteristics reported by Peace Corps volunteers in Timor-Leste by status of dengue virus infection, October 2018–June 2019 (*n* = 27)

Prevention measure/Characteristic	All participants, *n* = 27 (%)	Participants with evidence of DENV infection, *n* = 7 (%)	Participants without evidence of DENV infection, *n* = 20 (%)	*P* value*
Recalls mosquito bites	27 (100)	7 (100)	20 (100)	–
When most often bitten by mosquitoes†§				0.0520
Morning	1 (4)	0 (0)	1 (6)	
Afternoon	2 (8)	2 (33)	0 (0)	
Early evening	7 (29)	1 (17)	6 (33)	
Late evening	20 (42)	1 (17)	9 (50)	
Nighttime	3 (13)	2 (33)	1 (6)	
Don’t recall	1 (4)	0 (0)	1 (6)	
Screens and AC use				
Screens on doors in bedroom	2 (7)	1 (14)	1 (5)	0.4587
Screens on doors in home	1 (4)	1 (14)	0 (0)	0.2593
Screens on windows in bedroom	16 (59)	5 (71)	11 (55)	0.6618
Screens on windows in home‡§	3 (11)	1 (14)	2 (10)	1.0000
AC use in bedroom	1 (4)	1 (14)	0 (0)	0.2593
AC use in home†§	1 (4)	1 (14)	0 (0)	0.2692
Repellent use				0.2921
At least once a day	8 (30)	1 (14)	7 (35)	
Weekly or less often	4 (15)	0 (0)	4 (20)	
Whenever notices mosquitoes	13 (48)	6 (86)	7 (35)	
Never	1 (4)	0 (0)	1 (5)	
Don’t recall	1 (4)	0 (0)	1 (5)	
Bed net use				1.0000
Every night	25 (93)	7 (100)	18 (90)	
Less than monthly	1 (4)	0 (0)	1 (5)	
Never	1 (4)	0 (0)	1 (5)	
Permethrin use on clothes before arriving‡§	0 (0)	0 (0)	0 (0)	–
Permethrin use on clothes since arriving†‡	0 (0)	0 (0)	0 (0)	–
Wears long sleeve shirts	12 (44)	1 (14)	11 (55)	0.0914
Wears long pants	15 (56)	3 (43)	12 (60)	0.6618
Wears a hat	11 (41)	2 (29)	9 (45)	0.6618
Uses mosquito coils	2 (7)	1 (14)	1 (5)	0.4587
Uses insecticide aerosols	8 (30)	2 (29)	6 (30)	1.0000
Where most often bitten by mosquitoes†‡§				0.1650
At home	14 (56)	3 (50)	11 (58)	
While shopping/running errands	1 (4)	1 (17)	0 (0)	
During leisure activities	5 (20)	2 (33)	3 (16)	
Completed training on mosquito avoidance§	24 (89)	7 (100)	17 (85)	0.5453
Knew dengue transmitted by mosquitoes§	17 (63)	3 (43)	14 (70)	0.3648
Knew exposure to dengue in TL§	15 (56)	4 (57)	11 55)	1.0000

* *P* < 0.05 = statistically significant.

† Denominator excludes participants who did not respond to question or responded incorrectly (i.e., selected multiple responses when single response requested) to when most often bitten by mosquitoes (*n* = 3), AC use in home (*n* = 2), permethrin use on clothing since arriving (*n* = 1), where most often bitten by mosquitoes (*n* = 2).

‡ Denominator includes participants who answered “could not recall” screens on bedroom windows (*n* = 1), permethrin use on clothing before arriving (*n* = 2) and after arriving (*n* = 2), and where most often bitten by mosquitoes (*n* = 5).

§ Denominator includes participants who answered “could not recall” when most often bitten by mosquitoes (*n* = 1), repellant use (*n* = 1), screens on home windows (*n* = 1), permethrin use on clothing before arriving (*n* = 2) and after arriving (*n* = 2), where most often bitten by mosquitoes (*n* = 5), completed mosquito avoidance training (*n* = 3), knew dengue transmitted by mosquitoes (*n* = 2), and knew dengue exposure in TL (*n* = 7).

## DISCUSSION

The incidence of dengue among PCVs has been reported to follow the epidemic trends of regions and countries where PCVs work.^12^ Through the medical records review in this investigation, we observed that during a dengue epidemic in Timor-Leste, more than half of the PCVs in-country sought medical care for fever, and one-third had laboratory diagnostic evidence of dengue. Although none of the dengue patients met criteria for severe dengue, medical evacuation was required for two-thirds of PCVs with dengue, highlighting the importance of continued clinical training to identify and provide timely treatment of dengue patients in resource-limited settings. Although no specific mosquito avoidance behaviors were associated with DENV infection, all PCVs recalled being bitten by mosquitos and most reported being bitten most often at home, which emphasizes their continuous risk of for arboviral infections and the need to maintain and improve prevention measures.

By reviewing PCVs’ medical records, we found the expected symptoms of dengue, including warning signs for progression to severe dengue. As expected, rash was significantly more common in volunteers with dengue. Rash has been estimated to occur in 50–82% patients with dengue,^16–18^ and its time of presentation and characteristics can be of use in identifying patients with dengue in areas where other acute febrile illnesses are endemic.^19,20^

Along with fever, all dengue patients identified through the medical record review presented with myalgia, and self-reported myalgia was significantly higher among participants in the survey and blood sample collection with evidence of dengue. Myalgia is commonly reported among dengue patients, more often in adults, and it has been more frequently reported in DENV-1 and DENV-3 infections.^21,22^

To increase awareness and improve knowledge of dengue clinical manifestations and management, re-training was provided to all Peace Corps medical officers providing medical care to PCVs around the world, and annual dengue clinical management refreshers were recommended along with the CDC online Dengue Clinical Case Management.^23^ As appropriate clinical management of dengue patients can significantly reduce mortality,^24,25^ improvement of clinical diagnosis and management was one component of the WHO Global Strategy for Dengue Prevention and Control to reduce dengue case-fatality rate by at least 50% by 2020.^26^ Additionally, maintenance of a list of medical evacuation criteria (e.g., warning signs for severe dengue), with periodic review to ensure alignment with local resources and current guidelines, was recommended for all Peace Corps posts where dengue is endemic. This is especially important in posts with similar conditions to Timor-Leste, where there is an important limitation in immediate hospital services for PCVs.

Because all PCVs recalled mosquito bites during their time in Timor-Leste, continued implementation of mosquito bite prevention measures should be a priority to protect PCVs against dengue and other arboviruses in this population. Volunteers reported that they were most frequently bitten by mosquitoes at home, and 11 (41%) reported not having screens on their bedroom windows. In some settings, window screens have been associated with reduced infestations of *Aedes aegypti* mosquitoes and potential reduction in risk of DENV infection.^27–29^ CDC recommends that travelers to and residents of the tropics reside in homes with intact window screens to avoid DENV infection.^30^ The use of bed nets has been associated with protection from DENV infection in some studies.^31–33^ *Aedes* mosquitoes are more active during the day, but the range of activity can include early hours of the morning and evening when bed nets can help prevent *Aedes* mosquito bites. CDC recommends the use of a bed net for dengue prevention, and PCVs should be encouraged to continue this practice.

CDC also recommends frequent use of mosquito repellents to avoid mosquito bites and DENV infection, and reapplication according to label instructions.^30^ As only one-third of interviewed PCVs reported daily use of repellent, this demonstrates an opportunity to improve prevention measures among PCVs in Timor-Leste. The provision and use of permethrin to apply on clothes and other articles prior to PCV’s departure and on a regular basis while in-country could also improve protection of PCVs against mosquito bites. On completion of this investigation, the Peace Corps ensured that posts could provide permethrin spray for clothes.

Finally, CDC also recommends, when possible, residing in homes with air conditioning to avoid mosquito bites, as homes with air conditioning are typically well sealed and consequently are less accessible to mosquitos.^34^ Access to air conditioning for most PCVs is limited because of living conditions. Air conditioning use was only reported by one PCV in Timor-Leste; therefore, we were not able to assess its potential protection against DENV infection in this investigation nor the feasibility of improving access to air conditioning for PCVs in Timor-Leste. We did not assess other mosquito control measures taken by PCVs, such as frequent removal of standing water in domestic and peri-domestic environments. Peace Corps medical officers provided information on prevention practices during the outbreak, including mosquito breeding sites reduction; but the feasibility or successful implementation of this approach is unknown, because keeping water in open containers was reported as a common practice in Timor-Leste by PCVs.

The findings of this investigation are subject to some limitations. First, as the total participants in the survey was 27 out of the 35 PCVs in-country and only 11 dengue patients were identified, this investigation’s power to detect factors associated with DENV infection was limited. Second, as DENV transmission in Timor-Leste was ongoing for at least 6 months before the survey and blood sample collection was conducted, we cannot rule out seroreversion and consequent misclassification of some participants. The duration of detectability of anti-DENV IgM antibody is not well defined, including how it may differ by infecting DENV serotype, status of primary versus secondary DENV infection, and symptomatic versus asymptomatic infection.^35^ Also, given the period for which participants were asked questions regarding symptoms and preventive behavior, recall bias may be a limitation for the accuracy and completeness of the collected data, especially for detailed information on frequency of using prevention methods. To try to avoid bias in the participants responses toward the socially desirable behaviors, identifiable data from questionnaires was kept confidential, reviewed, and analyzed by CDC staff and not shared with Peace Corps leadership, and this was communicated to all participants before and during the questionnaire completion. As some desirable preventive behaviors like daily use of repellent were reported in less than half of PCVs in both groups, we do not expect the results of the questionnaire to be biased. Finally, although the recommendations regarding behavioral approaches to reduce exposure to mosquitos may be of use for PCVs in other areas of the tropics, and for other long-term care travelers, the specific findings of this investigation may not be generalizable to other PCVs or other long-term care travelers, because the specific characteristics of each group of travelers and each destination vary widely.

As the global burden of dengue has increased in recent years,^36,37^ so has the incidence of dengue among travelers to the tropics.^38^ This burden is expected to continue to grow in Asia, Sub-Saharan Africa, and Latin America, with global estimates of more than 50 million febrile illness cases and 2 million hospitalizations per year,^39^ further increasing the risk for travelers to these areas. Dengue infection risk in long-term travelers (more than 6 months) and business expatriates (those who reside in another country for occupational purposes, and will return to their country of origin after their assignment is completed) has been associated with increased duration of assignments and local epidemiology of dengue in the destination.^40,41^ As long-term travelers to dengue endemic areas with potential occurrence of outbreaks during their assignment of > 2 years, PCVs will continue to be at increased risk for dengue and other arboviral diseases.

Different than many other long-term travelers, PCVs undergo a complete medical assessment before their assignment and receive standardized training on prevention measures for different risks and conditions. Because most volunteers recalled completing training on mosquito avoidance before their departure, the inclusion of specific information on dengue and other arboviral diseases (chikungunya, Zika) in the training schedule may be beneficial. This approach of including relevant information on dengue risk factors could also be helpful for pre-travel counseling for other types of long-term travelers. An annual refresher on mosquito avoidance behavior and arboviral diseases is recommended for PCVs, along with refreshers when outbreaks are declared, and they should include the most common clinical presentation for dengue and key symptoms like fever, rash, and myalgia, as found in this report. The use of peer accounts and personal experiences from PCVs with dengue may provide a more impactful and lasting notion of the real effects and potential complications of dengue among incoming PCVs. Besides pre-travel training and annual refreshers, several mechanisms have been identified and have been used to communicate to PCVs or to host families crucial information on dengue outbreaks and prevention measures, including the use of newsletters, messages through group chats, discussions during medical care sought for other reasons, and inclusion of dengue prevention information during host families’ orientation.

DENV infection risk is determined by multiple factors, including socioeconomic, behavioral, and environmental factors. There is limited information available regarding the association between the nature of the work in volunteers and other aid workers and an increased risk of DENV infection,^40^ but some studies have found an increased risk of dengue with specific activities that might increase the risk for mosquito bites, like playing outdoors sports.^42^ Our investigation did not find any differences in DENV infection by type of work (education versus community economic development), work conditions (indoors versus outdoors), or mosquito avoidance behaviors. However, given the limitations of this investigation that include a small sample size, preventive behaviors should continue to be encouraged to decrease risk of DENV infection among PCVs. The results of this investigation and subsequent recommendations may also be of use to PCVs in other countries where dengue is endemic.

## Supplemental table

Supplemental materials
