# Supplementary material for: Dengue Virus Infections among Peace Corps Volunteers in Timor-Leste, 2018–2019
Source: Am J Trop Med Hyg. 2021 Apr 26;104(6):2202–9. doi: 10.4269/ajtmh.21-0020 (PMC8176509; doi:10.4269/ajtmh.21-0020)
Supplement: Supplementary file 1 [file tpmd210020.SD1.pdf]

Supplemental Table 1: Additional characteristics of Peace Corps Volunteers in Timor-Leste participanting in Survey and Blood Sample Collection by status of dengue virus infection, October 2018--June 2019 (n = 27)

	**All participants N = 27 (%)**	**Participants with evidence of DENV infection n = 7 (%)**	**Participants without evidence of DENV infection n = 20 (%)**	**p-value**
**Demographic characteristics**				
Age in years, median (range)	25 (22–67)	26 (23–45)	25 (22–67)	0.4210
Female sex, n (%)	10 (37)	4 (57)	6 (30)	0.3648
**Prior arbovirus exposures, n (%)**				
Previous residence in DENV endemic area	7 (26)	0 (0)	7 (35)	0.1369
Previous DENV diagnosis	2 (7)	2 (29)	0 (0)	0.0598
Vaccinated for Japanese encephalitis*	21 (78)	6 (86)	15 (75)	1.0000
Vaccinated for Yellow Fever*	7 (26)	1 (14)	6 (30)	0.6334
**Living and work conditions**				
Living situation:				1.0000
With host family, n (%)	26 (96)	7 (100)	19 (95)	
Alone, n (%)	1 (4)	0 (0)	1 (5)	
Municipality of residence:				0.2443
Liquiçá, n (%)	6 (22)	3 (43)	3 (15)	
Baucau, n (%)	3 (11)	0 (0)	3 (15)	
Bobonaro, n (%)	1 (4)	0 (0)	1 (5)	
Ermera, n (%)	5 (19)	0 (0)	5 (25)	
Aileu, n (%)	5 (19)	1 (14)	4 (20)	
Ainaro, n (%)	5 (19)	3 (43)	2 (10)	
Manufahi, n (%)	2 (7)	0 (0)	2 (10)	

Works and lives in same municipality, n (%)	27 (100)	7 (100)	20 (100)	--
Work type:				0.5799
Education, n (%)	5 (19)	2 (29)	3 (15)	
Community economic development, n (%)	22 (81)	5 (71)	17 (85)	
Work condition:				0.1751
Indoors only, n (%)	9 (33)	4 (57)	5 (25)	
Outdoors, n (%)	18 (67)	3 (43)	15 (75)	
Slept ≥1 night in municipality of: †**				
Oecusse, n (%)	1 (4)	0 (0)	1 (5)	1.0000
Liquiçá, n (%)	14 (54)	5 (71)	9 (47)	0.3913
Dili, n (%)	23 (88)	7 (100)	16 (84)	0.5396
Manatuto, n (%)	1 (4)	1 (14)	0 (0)	0.2692
Baucau, n (%)	9 (35)	2 (29)	7 (37)	1.0000
Lautém, n (%)	4 (15)	1 (14)	3 (16)	1.0000
Bobonaro, n (%)	4 (15)	2 (29)	2 (11)	0.2870
Ermera, n (%)	10 (38)	2 (29)	8 (42)	0.6680
Aileu, n (%)	10 (38)	2 (29)	8 (42)	0.6680
Viqueque, n (%)	0 (0)	0 (0)	0 (0)	--
Cova Lima, n (%)	1 (4)	0 (0)	1 (5)	1.0000
Ainaro, n (%)	14 (54)	5 (71)	9 (47)	0.3913
Manufahi, n (%)	6 (23)	1 (14)	5 (26)	1.0000
Nights slept outside of municipality of residence, median (range) †**	24 (0–271)	22 (7–74)	29 (0–271)	0.1934
Slept ≥1 night outside of municipality of residence at: †**				
Volunteers home, n (%)	16 (58)	5 (71)	10 (53)	0.6576

Family, n (%)	11 (42)	2 (29)	9 (47)	0.6576
Hostel, n (%)	17 (65)	4 (57)	13 (68)	0.6613
Hotel, n (%)	12 (46)	4 (57)	8 (42)	0.6652
Other, n (%)	6 (23)	2 (29)	4 (21)	1.0000

*Denominator includes participants who answered “could not recall” for Japanese encephalitis vaccination status (n = 5) and yellow fever vaccination status (n = 11).

†Between October 1, 2018 and July 19, 2019.

**Does not include individuals who did not respond to question or responded incorrectly (i.e., selected multiple responses when single response requested).
